# Sarcopenia Is Associated with High Pulse Pressure in Older Women

**DOI:** 10.1155/2015/109824

**Published:** 2015-08-05

**Authors:** Hélio José Coelho Júnior, Samuel da Silva Aguiar, Ivan de Oliveira Gonçalves, Ricardo Aurélio Carvalho Sampaio, Marco Carlos Uchida, Milton Rocha Moraes, Ricardo Yukio Asano

**Affiliations:** ^1^School of Physical Education, State University of Campinas, 130883851 Campinas, Brazil; ^2^Center of Health Sciences, University of Mogi das Cruzes, 08770-490 Mogi das Cruzes, Brazil; ^3^Graduate Program on Physical Education, Catholic University of Brasilia, 71966-700 Brasilia, Brazil; ^4^Community Center for Older People of Poá, Poá, Brazil; ^5^School of Sciences and Letters of Bragança Paulista, SP, Brazil

## Abstract

*Introduction*. Sarcopenia is a geriatric syndrome associated with impairment of muscle function, metabolism, and cognition in older women. Recent studies have shown a relationship between changes in muscle mass and the cardiovascular system. However, this relationship has not been fully elucidated. *Methods*. One hundred and thirty community-dwelling Brazilian older women (65.4 ± 6.3 years) were recruited to participate in this study. Data on body composition (via bioelectrical impedance measurements), cardiovascular parameters (using an automatic and noninvasive monitor), and muscle function (using a 3-meter gait speed test) were measured. *Results*. Sarcopenic older women (*n* = 43) presented higher levels of pulse pressure (PP) (60.3 ± 2.6 mmHg) and lower muscle function (0.5 ± 0.0 m/s) compared with nonsarcopenic subjects (*n* = 87) (53.7 ± 1.5 mmHg; 0.9 ± 0.0 m/s) (*P* < 0.05). Linear regression analysis demonstrated a significantly negative association between skeletal muscle index (SMI) and PP levels (*β* = −226, *P* < 0.05). Furthermore, sarcopenic older women showed a 3.1-fold increased risk of having higher PP levels compared with nonsarcopenic women (IC = 1.323–7.506) (*P* < 0.05). *Conclusion*. Sarcopenic older women showed lower muscle function and higher cardiovascular risk due to increased PP levels compared with nonsarcopenic subjects.

## 1. Introduction

Sarcopenia is a geriatric syndrome primarily characterized by progressive and generalized loss of skeletal muscle mass. In addition, it is strongly associated with the aging process and can be exacerbated in the presence of pathological conditions (e.g., diabetes mellitus type II) [1–5]. Sarcopenia negatively affects the autonomy and quality of life of older adults in different ways (e.g., by decreasing muscle strength and muscle power and increasing the risk of falls) [6]. In this respect, sarcopenia is associated with increased risk of falls and bone fractures, cognitive and muscle impairment, frailty, impaired performance of activities of daily living, loss of independency, and early death [1–5].

With regard to metabolic processes, older adults with lower skeletal muscle index (SMI) can present hyperinsulinemia, dyslipidemia, high levels of glycosylated hemoglobin, and predisposition to prediabetic and metabolic syndrome conditions, particularly if SMI is associated with obesity [7, 8]. Moreover, sarcopenic and nonsarcopenic older adults showed higher levels of insulin resistance and fasting glucose compared with nonsarcopenic older adults [4].

Although some studies demonstrated the relationship between morphofunctional and metabolic alterations due to sarcopenia, the effects of the decrease in skeletal muscle mass on the cardiovascular system are still inconclusive. Recent studies have reported that alterations in the SMI and increased arterial stiffness can negatively affect the cardiovascular system [9–11]. Ochi and colleagues [9] indicated that older adults with lower thigh muscle cross-sectional area presented higher levels of risk factors for cardiovascular disease, including increased intima-media thickness and increased pulse-wave velocity. Similarly, Abbatecola and colleagues [10] observed higher pulse-wave velocity in American older adults with lower SMI.

Pulse pressure (PP) is a simple and inexpensive measurement of arterial stiffness and is closely related to pulse-wave velocity [12, 13]. Furthermore, meta-analysis data indicate that increased PP is a good predictor of cardiovascular death and death from other causes in older people [14].

Therefore, in addition to the impairment of muscle function, autonomy, and metabolism caused by sarcopenia in older adults, recent studies showed a possible negative effect of sarcopenia on the cardiovascular system [4, 9]. Therefore, the aim of the present study was to evaluate the relationship between the sarcopenia index and hemodynamic dysfunctions in older women. The proposed hypothesis is that high levels of sarcopenia can negatively influence hemodynamic variables.

## 2. Methods

### 2.1. Study Design

This cross-sectional study evaluated 130 community-dwelling older women living in Brazil. All volunteers were recruited from two specialized healthcare centers for older women: (I) a community center for older adults and (II) Institute Reborn for seniors. In both centers, volunteers developed activities that involved predominantly the physical and cognitive domains. The physical activities offered were (1) a multimodal exercise program, which stimulates some physical capabilities (i.e., muscle strength, muscle power, and cardiorespiratory fitness) in the same session of exercise; these sessions lasted approximately 40 minutes and were performed in low-moderate intensity; (2) water aerobics; (3) folk dance; and (4) yoga. Painting was the only activity that involved predominantly the cognitive domain. After registration, older adults were automatically enrolled in all activities. However, they were not required to participate in or perform activities every day.

Participation in the present study involved the completion of all measurements after signing the informed consent form. This study was approved by the Research Ethics Committee of the Universidade de Mogi das Cruzes (UMC) under protocol number 621-614. This study was developed in accordance with the Declaration of Helsinki and according to Resolution 196/96 of the National Health Council.

### 2.2. Participants

All older women were ≥60 years of age. The exclusion criteria were use of hormone replacement and/or psychotropic drugs, cardiovascular disease (e.g., acute myocardial infarction, stroke, peripheral arterial disease, and transient ischemic disease), pulmonary disease, neurological or psychiatric disease (e.g., Parkinson's or Alzheimer's disease), musculoskeletal disorders, and comorbidities associated with greater risk of falls. All clinical information (i.e., disease status, age, and drug therapy) was obtained by reviewing the medical records of each subject.

All subjects were instructed to refrain from physical exercise for 96 hours before the tests and to refrain from eating or drinking (including water) for 8 hours before the tests. All tests were conducted between 07:00 am and 10:00 am under a controlled temperature of 26°C.

### 2.3. Measurements

#### 2.3.1. Assessment of Body Composition and Sarcopenia

A data acquisition system (Tanita InnerScan 50v, Tokyo, Japan) was used to measure the bioelectrical impedance. This system uses an electrical current to quantify the amount of intracellular and extracellular water in the body. The device has four electrodes: two are placed on the feet while the other two are placed on the hands of the volunteers. The device measures body mass index (BMI), total body mass, total muscle mass, and percentage of fat mass [15]. Sarcopenia was defined by the criterion of Janssen and colleagues [16]. In summary, the bioelectrical impedance test allows the normalization of absolute muscle mass (AMM) to height (AMM/height^2^), which is denominated skeletal muscle mass index (SMI). Sarcopenia was classified into tertiles [11]. Older women classified into the first tertile (<15, 68 kg/m^2^) were classified as sarcopenic.

#### 2.3.2. Cardiovascular Parameters

All cardiovascular parameters were analyzed at rest. The procedures for the measurement of blood pressure were adapted from the VII Joint National Committee of High Blood Pressure (JNC7) [17]. In summary, older women remained in the sitting position in a comfortable chair for 15 minutes in a dark and quiet room. After this period, a cuff with the right size was placed approximately at the midpoint of the upper left arm (heart level). An automatic, noninvasive and validated [18] arterial blood pressure monitor (Microlife-BP 3BT0A, Microlife, Widnau, Switzerland) was used to measure systolic blood pressure (SBP), diastolic blood pressure (DBP), and heart rate (HR). The mean arterial pressure (MAP), rate pressure product (RPP), and pulse pressure (PP) were evaluated according to the following equations: MAP = [SBP + (2 ∗ DBP)]/3; RPP = SBP ∗ HR; PP = SBP − DBP [12, 19]. The size of the arm cuff was selected after measuring the arm circumference (Sanny, São Paulo, Brazil).

#### 2.3.3. Muscle

Muscle function was evaluated using the 3-meter walk speed test (WS) test. During the test, the volunteers were instructed to walk a distance of 5 meters at a normal cadence. To ensure this, the researcher requested the following: “Please, walk as you were going to a supermarket or bakehouse.” Volunteers should remain with both feet on the starting line and counting started when one foot touched the 1-meter line and stopped when one foot touched the 4-meter line. After a 1-minute rest, a second attempt was made. The mean values were used in the analysis [15].

#### 2.3.4. Pathological Conditions and Cofactors

Medical records were reviewed to obtain information of the health status of each volunteer. The head physician recorded the pathological conditions but a specialist not affiliated to/outside the center made the pathologic diagnosis. The cofactors (the number of drugs used by older women (mean of drugs) and smoking status) were recorded by a head nurse after interviewing the participants. The medical records were updated every six months.

### 2.4. Statistical Analysis

The Shapiro-Wilk test was used to calculate data normality. Student's *t*-test was used to compare quantitative variables and *χ* ^2^ was used to compare qualitative variables. Pearson's correlations were conducted with and without adjusting for pathological conditions. After statistical analyses, it was verified that PP values were higher in the sarcopenic group and were significantly associated with SMI; subsequently, the correlation between SMI and PP was investigated. Stepwise linear regression was conducted to predict the best model to evaluate the association between SMI and PP. Multiple linear regression analysis was used to assess the significance of the proposed model. To assess whether the IMM was a risk factor for PP, logistic regression was conducted. Differences were considered statistically significant at *P* < 0.05. Sample size was determined on the basis of a population of 112.481 individuals and a confidence level of 95%. All analyses were conducted using the Statistical Package for the Social Sciences (SPSS; IBM, Chicago, IL, USA) software version 20.0.

## 3. Results

Subjects were dichotomized in two groups according to the SMI cutoff.  Table 1 presents the characteristics of the study group. Sarcopenic older women presented lower values of BMI, SMI, and AMM and lower muscle function but higher PP values compared with normal older women.

Pearson's correlation was performed to assess possible associations between the cardiovascular variables and SMI. Our results showed a significantly negative association between SMI and PP (−0.25; *P* = 0.03). Moreover, adjustment for pathological conditions alone or in combination did not change the significance of the association (hypertension (−0.25; *P* = 0.05); diabetes mellitus type II (−0.25; *P* = 0.05); metabolic syndrome (−0.25; *P* = 0.04); respiratory pathology (−0.25; *P* = 0.05); and metabolic syndrome together with respiratory pathology (−0.25; *P* = 0.04)).

Table 2 shows data on linear and multiple linear regression. The findings on linear regression showed a significant *R* ^2^ (0.49) for IMM in relation to PP. However, multiple regression did not show significant results for any of the proposed models from stepwise linear regression.

The odds ratio results indicate that older women with sarcopenia have three times increased risk (3.151) of having increased PP values compared with nonsarcopenic volunteers (1.323–7.506 (95% confidence interval), *P* < 0.05).

## 4. Discussion

The results of the present study indicate that sarcopenic older women have lower muscle function and higher PP levels compared with nonsarcopenic older women. These results were confirmed by using multilinear and linear regression and indicate a significantly negative association between SMI and PP. Furthermore, sarcopenic volunteers had a three times higher risk of having increased PP values compared with nonsarcopenic individuals.

These findings are in accordance with those of other studies, which demonstrated a relationship between the sarcopenia index and cardiovascular alterations in older adults [4, 9–11, 20]. Recently, Han and colleagues [4] evaluated a large sample of older Koreans (*n* = 4.846) and observed a higher prevalence of hypertension in sarcopenic older adults compared with nonsarcopenic adults, regardless of the obesity level. In addition, nonobese sarcopenic older adults had a 1.5 times higher risk of presenting with hypertension compared with nonsarcopenic subjects.

Furthermore, other experiments have investigated whether sarcopenia could be associated with increased arterial stiffness, which can promote the development of hypertension as well as other cardiovascular and cerebrovascular diseases [12, 21–23]. It has also been demonstrated that increased PP levels are an important risk factor for coronary heart disease [18].

Similar to the results of the present study, other studies demonstrated an association between the degree of sarcopenia and arterial stiffness. These experiments observed that increase in sarcopenia was associated with increase in brachial-ankle pulse-wave velocity in Japanese [7, 11, 20] and American older adults [10]. Furthermore, Srikanthan and Karlamangla [7] observed that the sarcopenia index was negatively associated with carotid intima-media thickness, suggesting a possible correlation with the formation of atherosclerotic plaques.

In the present study, older women with lower SMI showed higher odds ratio for higher PP levels compared with nonsarcopenic women. Higher levels of brachial PP are associated with increased pulse-wave velocity, which is mainly due to the decrease large-vessel compliance [12]. Complications associated with higher PP values have been previously studied [21–23]. In normotensive and hypertensive populations, higher PP was the most important determinant of the risk of death (mainly due to cardiovascular complications) [21, 22]. For this reason, the possibility of using PP as a better predictor of cardiovascular death compared with other hemodynamic measurements (e.g., SBP and DBP) has been recently considered [23].

One of the limitations of the present study was the failure to assess the influence of pathological conditions on the association between SMI and PP. However, no changes were observed in our results using Pearson's correlation and multilinear regression after adjusting for morbidities. Diabetes type II (DMTII) seems to enhance the loss of muscle mass primarily in individuals with uncontrolled glycemia [5–24]. Furthermore, patients with low SMI with and without DMTII demonstrated increased insulin resistance, hyperinsulinemia, and glycosylated hemoglobin levels compared with individuals with higher SMI [7]. Recent evidence suggests a potential correlation between sarcopenia and dyslipidemia, which leads to hyperglycemia and ultimately metabolic syndrome [8]. However, these data were not confirmed in the present study.

It is possible that the number of volunteers with pathologies was insufficient to evaluate the impact of each condition on the parameters evaluated. However, an advantage of the limited number of older people with illnesses is that, even in healthy older women, decreased muscle mass can impair the cardiovascular system.

A possible mechanism that could explain the association between SMI and higher PP levels in the sarcopenic group is increased chronic inflammation due to decreased muscle function, which can lead to physical inactivity. Decreased muscle function resulted in higher levels of inflammatory markers in the blood of older adults [25]. Chronic low-grade inflammation is characterized by an increase in blood levels and gene expression of proinflammatory cytokines, which can—via paracrine and autocrine mechanisms—decrease the bioavailability of nitric oxide, increase arterial stiffness, increase the formation of atherosclerotic plaques, and promote endothelial dysfunction, which, in turn, can promote an increase in PP levels [26–28].

On the other hand, the increase in muscle activity has been suggested as a mechanism that can inhibit chronic low-grade inflammation [29]. It is known that bioactive proteins known as myokines (e.g., IL-6) are produced by muscle fibers from muscle contraction [30, 31]. Myokines can inhibit the activity of proinflammatory cytokines (e.g., TNF-*α*), thereby avoiding the deleterious effect of cytokines on the human body [25, 27]. Animal experiments showed that the increase in muscle activity by physical exercise could decrease the activity of TNF-*α* in mice that overexpress this protein [32]. In addition, the cytokine levels of volunteers who practiced physical exercise in the same day and who were stimulated to produce TNF-*α* did not increase. However, the group that did not practice physical exercise had increased TNF-*α* concentrations [33]. In the present study, nonsarcopenic older women with improved muscle function possibly performed more muscle contractions, which led to the increased production of myokines, which in turn acted as a protective factor against chronic low-grade inflammation.

Insulin resistance can also explain the results of this study. Individuals with low SMI with and without diabetes demonstrated increased insulin resistance, hyperinsulinemia, and increased levels of glycosylated hemoglobin compared with individuals with higher SMI, which was probably caused by decreased insulin levels in the target organ [7]. Furthermore, hyperinsulinemia due to insulin resistance can activate NF*κ*B, which is an important regulator of the production of proinflammatory cytokines [34]. Clearly, a synergistic effect of both mechanisms cannot be discarded and is probably the best explanation.

Interestingly, the cofactors identified in the present study (BMI, percentage of body fat, age, and muscle function) had no effect on the association between SMI and PP. Other factors that can influence vascular homeostasis, such as nitric oxide, cholesterol, and low- and high-density lipoproteins, can be used to create a better model to investigate the relationship between SMI and PP. Moreover, studies involving study groups with specific pathologic conditions, such as DMT2, hypertension, obesity, and metabolic syndrome, can help elucidate the impact of pathological conditions on muscle mass homeostasis and their effect on the cardiovascular system [5, 8].

Some of the limitations of the present study include (i) its cross-sectional design, which precluded the assessment of a time-effect relationship; (ii) the limited number of volunteers enrolled, which can be partially explained by sample homogeneity, which contributed to a better understanding of the effect of sarcopenia on older women; and (iii) the absence of biochemical measurements, which could have elucidated possible interacting mechanisms. However, to the best of our knowledge, this is the first study that addressed the relationship between low SMI and changes in the arterial stiffness index in Brazilian older women.

## 5. Conclusion

The present study showed that older women with higher SMI (which is predominantly influenced by an active lifestyle) presented lower PP levels and decreased cardiovascular risk whereas older women with a sarcopenic profile had a cardiovascular risk 3 times higher than that of nonsarcopenic women. Therefore, beneficial therapies, such as strength exercise, can help increase or maintain muscle mass and consequently improve the quality of life of older people.

## Figures and Tables

**Table 1:** Comparison between the groups with regard to morphological and functional variables and health indicators.

	Healthy patients (*n* = 87)	Sarcopenic patients (*n* = 43)	*P*
Age (years)	64.1 ± 0.8	64.5 ± 0.5	ns
Body mass (kg)	66.2 ± 1.0	66.3 ± 2.5	ns
Height (cm)	155.9 ± 0.0	156.8 ± 0.0	ns
Body mass index (kg/m^2^)	28.1 ± 0.4	24.3 ± 0.5	<0.05
Absolute muscle mass (kg)	41.5 ± 0.4	34.9 ± 1.3	<0.05
Skeletal muscle index (kg/m^2^)	17.4 ± 0.1	14.6 ± 0.1	<0.05
Fat mass (%)	34.8 ± 0.8	36.4 ± 1.7	ns
SBP (mmHg)	138.8 ± 0.0	144 ± 0.1	ns
DBP (mmHg)	84.3 ± 1.1	83.4 ± 2.1	ns
MAP (mmHg)	102.6 ± 1.3	103.7 ± 2.1	ns
HR (bpm)	75.7 ± 1.2	74.9 ± 1.7	ns
RPP (mmHg·bpm)	10530 ± 256.7	10750 ± 303.9	ns
PP (mmHg)	53.7 ± 1.5	60.3 ± 2.6	<0.05
Functionality (m/s)	0.9 ± 0.0	0.5 ± 0.0	<0.05
Hypertension (%)	41.0	65.0	ns
Diabetes mellitus type II (%)	4.4	5.8	ns
Metabolic syndrome (%)	4.0	4.0	ns
Respiratory pathology (%)	4.0	1.0	ns
Mean of drugs	1.1 ± 0.1	1.7 ± 0.2	ns
Smoker (%)	5.0	5.0	ns

Data are presented as mean ± SE. SBP = systolic blood pressure; DBP = diastolic blood pressure; MAP = mean arterial pressure; HR = heart rate; RPP = rate pressure product; PP = pulse pressure.

**Table 2:** Linear and multilinear regression considering pulse pressure as the dependent variable.

Variable(s)	*R* ^2^	*β*	*P*
SMI	0.49	−0.226	<0.05
Model 1	0.94	0.157	ns
Model 2	0.136	0.104	ns
Model 3	0.174	0.98	ns

SMI = skeletal muscle index; Model 1 = SMI plus age; Model 2 = Model 1 plus metabolic syndrome, smoking status, hypertension, and diabetes mellitus type II; Model 3 = Model 2 plus height and functionality.
